# 8-Hy­droxy­quinolin-1-ium hydrogen sulfate monohydrate

**DOI:** 10.1107/S1600536813022319

**Published:** 2013-08-21

**Authors:** Maamar Damous, George Dénès, Sofiane Bouacida, Meriem Hamlaoui, Hocine Merazig, Jean-Claude Daran

**Affiliations:** a Unité de Recherche de Chimie de l’Environnement et Moléculaire Structurale, CHEMS, Université Constantine 1, 25000, Algeria; b Laboratory of Solid State Chemistry and Mössbauer Spectroscopy, Laboratories for Inorganic Materials, Department of Chemistry and Biochemistry, Concordia University, Montreal, Quebec, H3G 1M8, Canada; c Département Sciences de la Matière, Faculté des Sciences Exactes et Sciences de la Nature et de la Vie, Université Oum El Bouaghi 04000, Algeria; d Laboratoire de Chimie de Coordination, UPR CNRS 8241, 205 route de Narbonne, 31077 Toulouse Cedex, France

## Abstract

In the crystal structure of the title salt hydrate, C_9_H_8_NO^+^·HSO_4_^−^·H_2_O, the quinoline N—H atoms are hydrogen bonded to the bis­ulfate anions. The bis­ulfate anions and water mol­ecules are linked together by O—H⋯O hydrogen-bonding inter­actions. The cations and anions form separate layers alternating along the *c* axis, which are linked by N—H⋯O and O—H⋯O hydrogen bonds into a two-dimensional network parallel to (100). Further O—H⋯O contacts connect these layers, forming a three-dimensional network, in which two *R*_4_^4^(12) rings and *C*_2_^2^(13) infinite chains can be identified.

## Related literature

For background to and the biological activity of quinoline derivatives, see: Sasaki *et al.* (1998 ▶); Reux *et al.* (2009 ▶); Morimoto *et al.* (1991 ▶); Markees *et al.* (1970 ▶). For related structures, see: Loh *et al.* (2010*a* ▶,*b* ▶). For a description of the Cambridge Structural Database, see: Allen, (2002 ▶).
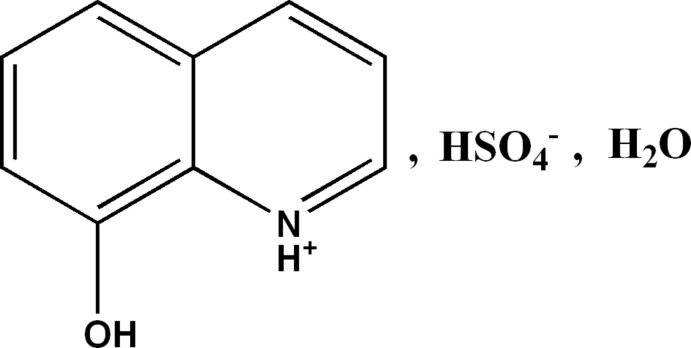


## Experimental

### 

#### Crystal data

C_9_H_8_NO^+^·HSO_4_^−^·H_2_O
*M* *_r_* = 261.25
Triclinic, 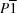

*a* = 6.5536 (4) Å
*b* = 8.0600 (5) Å
*c* = 11.3369 (6) Å
α = 100.068 (5)°
β = 106.344 (4)°
γ = 105.712 (5)°
*V* = 532.35 (5) Å^3^
*Z* = 2
Mo *K*α radiation
μ = 0.32 mm^−1^
*T* = 180 K
0.43 × 0.16 × 0.08 mm

#### Data collection

Agilent Xcalibur (Sapphire1) diffractometer
Absorption correction: multi-scan (*CrysAlis PRO*; Agilent, 2011 ▶) *T*_min_ = 0.874, *T*_max_ = 0.975
10891 measured reflections
2171 independent reflections
2000 reflections with *I* > 2σ(*I*)
*R*_int_ = 0.027

#### Refinement

*R*[*F* ^2^ > 2σ(*F* ^2^)] = 0.030
*wR*(*F* ^2^) = 0.079
*S* = 1.05
2171 reflections
163 parameters
3 restraints
H atoms treated by a mixture of independent and constrained refinement
Δρ_max_ = 0.41 e Å^−3^
Δρ_min_ = −0.39 e Å^−3^

### 

Data collection: *CrysAlis PRO* (Agilent, 2011 ▶); cell refinement: *CrysAlis PRO*; data reduction: *CrysAlis PRO*; program(s) used to solve structure: *SIR2002* (Burla *et al.*, 2003 ▶); program(s) used to refine structure: *SHELXL97* (Sheldrick, 2008 ▶); molecular graphics: *ORTEP-3 for Windows* (Farrugia, 2012 ▶) and *DIAMOND* (Brandenburg & Berndt, 2001 ▶); software used to prepare material for publication: *WinGX* (Farrugia, 2012 ▶).

## Supplementary Material

Crystal structure: contains datablock(s) I. DOI: 10.1107/S1600536813022319/hg5339sup1.cif

Structure factors: contains datablock(s) I. DOI: 10.1107/S1600536813022319/hg5339Isup2.hkl

Supplementary material file. DOI: 10.1107/S1600536813022319/hg5339Isup3.cml

Additional supplementary materials:  crystallographic information; 3D view; checkCIF report

## Figures and Tables

**Table 1:** Hydrogen-bond geometry (Å, °)

*D*—H⋯*A*	*D*—H	H⋯*A*	*D*⋯*A*	*D*—H⋯*A*
N1—H1⋯O14	0.88	2.00	2.7690 (18)	145
O1*W*—H1*W*⋯O11^i^	0.85 (1)	1.89 (1)	2.7369 (17)	178 (1)
O1*W*—H2*W*⋯O14^ii^	0.85 (1)	2.03 (1)	2.8818 (19)	175 (1)
O9—H9⋯O13^iii^	0.84	1.81	2.6470 (16)	174
O12—H12⋯O1*W*	0.84	1.72	2.5529 (17)	172

Symmetry codes: (i) 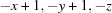; (ii) 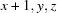; (iii) 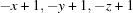.

**Fig. 1.**
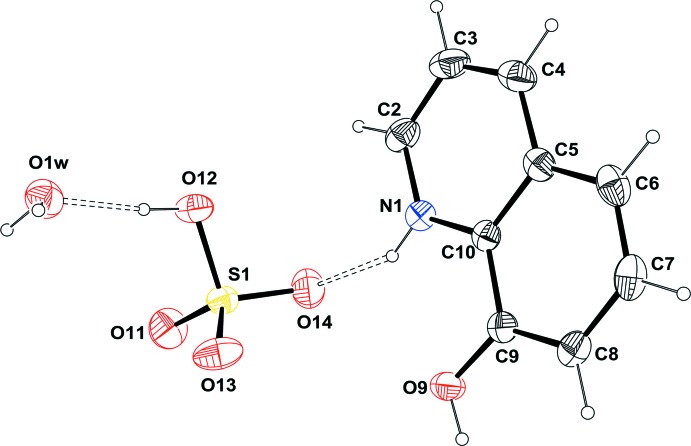
(Farrugia, 2012) The molecule structure of the title Compound with the atomic labelling scheme·Displacement are drawn at the 50% probability level. H atoms are represented as small spheres of arbitrary radius.

**Fig. 2.**
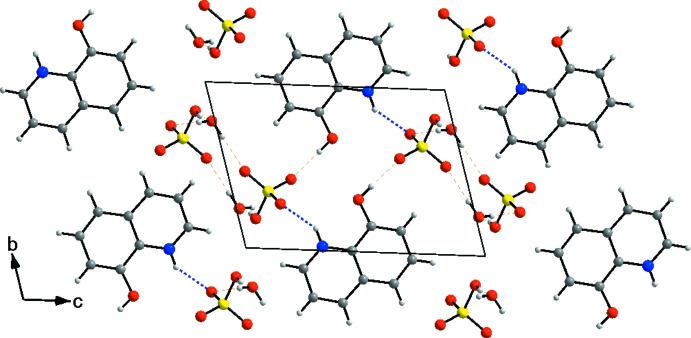
(Brandenburg & Berndt, 2001) A diagram of the layered crystal packing in (I), viewed down the a axis showing hydrogen bond as dashed line.

**Fig. 3.**
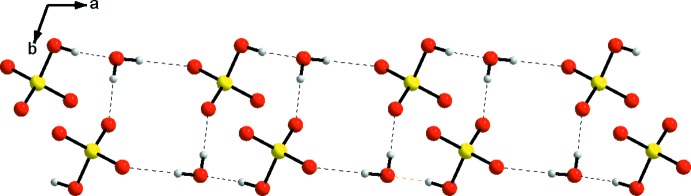
(Brandenburg & Berndt, 2001) Hydrogen bond connections as dashed line between hydrogenesulfate and water molecule building chain along the a axis.

## supplementary crystallographic information

### 1. Comment

Recently, hydrogen-bonding patterns involving quinoline and its derivatives with organic acid have been investigated (Loh *et al.*, 2010*a*,*b*). Syntheses of the quinoline derivatives were discussed earlier (Sasaki *et al.*, 1998; Reux *et al.*, 2009). Quinolines and their derivatives are very important compounds because of their wide occurrence in natural products (Morimoto *et al.*, 1991) and biologically active compounds (Markees *et al.*, 1970). Herein we report the synthesis and crystal structure of 8-hydroxy-quinolin-1-ium hydrogen sulfate monohydrate (I). The molecular structure of (I), and the atomic numbering used, is illustrated in Fig. 1. The asymmetric unit of (I) consists of consists of one 8-hydroxy-quinolin-1-ium cation, one hydrogen sulfate anion and one water molecule. One proton is transferred from the hydroxyl group of sulfuric acid to the atom N1 of 8-hydroxy-quinoline during the crystallization, resulting in the formation of salt. All bond distances and angles are within the ranges of accepted values (CSD, Allen, 2002).

In the crystal structure, cationic and anionic layers alternate along the *c* axis and are linked by intermolecular N—H···O and O—H···O hydrogen bonds resulting in a two-dimensional network parallel to (100) (Table 1; Fig.2). Further O—H···O contacts connect these layers, forming a three-dimensional network in which *R*^4^_4_(12) rings and C^2^_2_(13) infinite chains are generated.

Perhaps the most interesting aspect of the structure results from the hydrogen bonding between the bisulfate anions and the solvent water molecule. This results in the formation of a ladder motif that runs parallel to the *a* axis (Fig. 3). Each bisulfate ion serves as a hydrogen bond donor to one water molecule and a hydrogen bond acceptor from a second water molecule forming the rails of the ladder, of form C^2^_2_(6). The rungs are formed *via* a second water-donor/bisulfate acceptor pair, which generates rings within the ladder structure (two rungs and two rail sections in each ring), *R*^4^_4_(12). There are two chemically different rings formed in this case since one involves rail sections with water molecules serving as the hydrogen bond donor and the other involves the bisulfate ion serving as the hydrogen bond donor.

### 2. Experimental

Single crystals of the compound C_9_H_7_NO^+^. HSO^-4^. H_2_O were grown as follows: 1 mmol of the copper sulfate pentahydrate CuSO_4_. 5H_2_O; 1 mmol of 8-hydroxy-quinoline and 1 mmol of sulfuric acid H_2_SO_4_ were mixed together in a minimum amount of distilled water. The clear solutions were stirred for 15 min and allowed to stand at room temperature. After a few days, Yellow crystals were formed. The product was filtered off, washed with a small amount of distilled water and was carefully isolated under polarizing microscope for analysis by X-ray diffraction.

### 3. Refinement

Hydrogen atoms were localized on Fourier maps but introduced in calculated positions and treated as riding on their parent atoms (C, O and N) with C—H= 0.95 Å, N—H= 0.88 Å and O—H= 0.84 Å and *U*_iso_(H)=1.2*U*_eq_(C or N); *U*_iso_(H)=1.5*U*_eq_(O). Exept for H1W and H2W atoms of water molecule were located in difference Fourier maps and included in the subsequent refinement with *U*_iso_(H) = 1.5*U*_eq_(O).

### Crystal data

**Table:**

C_9_H_8_NO^+^·HSO_4_^−^·H_2_O	*Z* = 2
*M**_r_* = 261.25	*F*(000) = 272
Triclinic, *P*1	*D*_x_ = 1.63 Mg m^−^^3^
*a* = 6.5536 (4) Å	Mo *K*α radiation, λ = 0.71073 Å
*b* = 8.0600 (5) Å	Cell parameters from 8303 reflections
*c* = 11.3369 (6) Å	θ = 3.3–28.4°
α = 100.068 (5)°	µ = 0.32 mm^−^^1^
β = 106.344 (4)°	*T* = 180 K
γ = 105.712 (5)°	Box, yellow
*V* = 532.35 (5) Å^3^	0.43 × 0.16 × 0.08 mm

### Data collection

**Table:**

Agilent Xcalibur (Sapphire1) diffractometer	2171 independent reflections
Radiation source: fine-focus sealed tube	2000 reflections with *I* > 2σ(*I*)
Graphite monochromator	*R*_int_ = 0.027
Detector resolution: 8.2632 pixels mm^-1^	θ_max_ = 26.4°, θ_min_ = 3.3°
ω scans	*h* = −8→8
Absorption correction: multi-scan (*CrysAlis PRO*; Agilent, 2011)	*k* = −10→10
*T*_min_ = 0.874, *T*_max_ = 0.975	*l* = −14→14
10891 measured reflections	

### Refinement

**Table:**

Refinement on *F*^2^	Secondary atom site location: difference Fourier map
Least-squares matrix: full	Hydrogen site location: inferred from neighbouring sites
*R*[*F*^2^ > 2σ(*F*^2^)] = 0.030	H atoms treated by a mixture of independent and constrained refinement
*wR*(*F*^2^) = 0.079	*w* = 1/[σ^2^(*F*_o_^2^) + (0.0355*P*)^2^ + 0.3301*P*] where *P* = (*F*_o_^2^ + 2*F*_c_^2^)/3
*S* = 1.05	(Δ/σ)_max_ < 0.001
2171 reflections	Δρ_max_ = 0.41 e Å^−^^3^
163 parameters	Δρ_min_ = −0.39 e Å^−^^3^
3 restraints	Extinction correction: *SHELXL97* (Sheldrick, 2008), Fc^*^=kFc[1+0.001xFc^2^λ^3^/sin(2θ)]^-1/4^
Primary atom site location: structure-invariant direct methods	Extinction coefficient: 0.064 (4)

### Special details

**Table:**

Experimental. Empirical absorption correction using spherical harmonics, implemented in SCALE3 ABSPACK scaling algorithm. CrysAlisPro (Agilent Technologies, 2011)
Geometry. All e.s.d.'s (except the e.s.d. in the dihedral angle between two l.s. planes) are estimated using the full covariance matrix. The cell e.s.d.'s are taken into account individually in the estimation of e.s.d.'s in distances, angles and torsion angles; correlations between e.s.d.'s in cell parameters are only used when they are defined by crystal symmetry. An approximate (isotropic) treatment of cell e.s.d.'s is used for estimating e.s.d.'s involving l.s. planes.
Refinement. Refinement of *F*^2^ against ALL reflections. The weighted *R*-factor *wR* and goodness of fit *S* are based on *F*^2^, conventional *R*-factors *R* are based on *F*, with *F* set to zero for negative *F*^2^. The threshold expression of *F*^2^ > σ(*F*^2^) is used only for calculating *R*-factors(gt) *etc*. and is not relevant to the choice of reflections for refinement. *R*-factors based on *F*^2^ are statistically about twice as large as those based on *F*, and *R*- factors based on ALL data will be even larger.

### Fractional atomic coordinates and isotropic or equivalent isotropic displacement parameters (Å^2^)

**Table:**

	*x*	*y*	*z*	*U*_iso_*/*U*_eq_	
C2	0.0574 (3)	−0.1138 (2)	0.22102 (15)	0.0278 (3)	
H2	0.0132	−0.0941	0.1388	0.033*	
C3	0.0242 (3)	−0.2869 (2)	0.23249 (16)	0.0322 (4)	
H3	−0.0426	−0.3858	0.1584	0.039*	
C4	0.0881 (3)	−0.3137 (2)	0.35064 (17)	0.0295 (4)	
H4	0.0666	−0.432	0.3587	0.035*	
C5	0.1857 (2)	−0.1681 (2)	0.46135 (15)	0.0227 (3)	
C6	0.2554 (3)	−0.1860 (2)	0.58640 (16)	0.0284 (3)	
H6	0.2366	−0.3013	0.6001	0.034*	
C7	0.3500 (3)	−0.0372 (2)	0.68755 (15)	0.0286 (4)	
H7	0.3962	−0.0503	0.7717	0.034*	
C8	0.3810 (2)	0.1351 (2)	0.67071 (14)	0.0248 (3)	
H8	0.4469	0.2363	0.7432	0.03*	
C9	0.3169 (2)	0.15841 (19)	0.55040 (14)	0.0204 (3)	
C10	0.2174 (2)	0.00521 (19)	0.44474 (13)	0.0193 (3)	
N1	0.1504 (2)	0.02392 (17)	0.32431 (11)	0.0213 (3)	
H1	0.1697	0.1324	0.3146	0.026*	
O1W	0.6635 (2)	0.23721 (17)	0.01309 (13)	0.0362 (3)	
H1W	0.686 (4)	0.329 (2)	−0.015 (2)	0.054*	
H2W	0.788 (2)	0.246 (3)	0.0684 (18)	0.054*	
O9	0.34231 (19)	0.31692 (14)	0.52328 (10)	0.0270 (3)	
H9	0.402	0.3998	0.5915	0.04*	
O11	0.2516 (2)	0.46432 (17)	0.07413 (12)	0.0396 (3)	
O12	0.30658 (19)	0.17896 (15)	0.07153 (11)	0.0303 (3)	
H12	0.4287	0.2071	0.0572	0.045*	
O13	0.4983 (2)	0.42607 (17)	0.26039 (11)	0.0384 (3)	
O14	0.0966 (2)	0.27484 (17)	0.19066 (13)	0.0371 (3)	
S1	0.28858 (6)	0.34687 (5)	0.15279 (3)	0.02286 (14)	

### Atomic displacement parameters (Å^2^)

**Table:**

	*U*^11^	*U*^22^	*U*^33^	*U*^12^	*U*^13^	*U*^23^
C2	0.0232 (7)	0.0338 (8)	0.0216 (7)	0.0084 (6)	0.0056 (6)	0.0013 (6)
C3	0.0266 (8)	0.0275 (8)	0.0324 (9)	0.0050 (6)	0.0078 (7)	−0.0056 (7)
C4	0.0244 (8)	0.0212 (7)	0.0424 (10)	0.0075 (6)	0.0133 (7)	0.0051 (7)
C5	0.0172 (7)	0.0231 (7)	0.0309 (8)	0.0085 (6)	0.0107 (6)	0.0089 (6)
C6	0.0255 (8)	0.0303 (8)	0.0385 (9)	0.0140 (6)	0.0141 (7)	0.0193 (7)
C7	0.0239 (8)	0.0431 (9)	0.0263 (8)	0.0161 (7)	0.0101 (6)	0.0177 (7)
C8	0.0197 (7)	0.0319 (8)	0.0204 (7)	0.0078 (6)	0.0060 (6)	0.0044 (6)
C9	0.0157 (7)	0.0225 (7)	0.0231 (7)	0.0065 (5)	0.0070 (5)	0.0055 (6)
C10	0.0146 (6)	0.0227 (7)	0.0212 (7)	0.0067 (5)	0.0066 (5)	0.0059 (6)
N1	0.0197 (6)	0.0226 (6)	0.0208 (6)	0.0073 (5)	0.0061 (5)	0.0055 (5)
O1W	0.0411 (7)	0.0315 (7)	0.0434 (8)	0.0157 (6)	0.0188 (6)	0.0159 (6)
O9	0.0332 (6)	0.0193 (5)	0.0230 (5)	0.0053 (4)	0.0067 (5)	0.0033 (4)
O11	0.0560 (8)	0.0384 (7)	0.0367 (7)	0.0252 (6)	0.0170 (6)	0.0241 (6)
O12	0.0311 (6)	0.0242 (6)	0.0311 (6)	0.0096 (5)	0.0086 (5)	−0.0001 (5)
O13	0.0405 (7)	0.0353 (7)	0.0282 (6)	0.0176 (6)	−0.0021 (5)	−0.0028 (5)
O14	0.0411 (7)	0.0367 (7)	0.0459 (7)	0.0182 (6)	0.0242 (6)	0.0187 (6)
S1	0.0289 (2)	0.0205 (2)	0.0203 (2)	0.01131 (15)	0.00646 (15)	0.00689 (14)

### Geometric parameters (Å, º)

**Table:**

C2—N1	1.324 (2)	C8—H8	0.95
C2—C3	1.387 (2)	C9—O9	1.3437 (18)
C2—H2	0.95	C9—C10	1.411 (2)
C3—C4	1.360 (3)	C10—N1	1.3602 (19)
C3—H3	0.95	N1—H1	0.88
C4—C5	1.410 (2)	O1W—H1W	0.849 (9)
C4—H4	0.95	O1W—H2W	0.853 (9)
C5—C6	1.408 (2)	O9—H9	0.84
C5—C10	1.410 (2)	O11—S1	1.4310 (12)
C6—C7	1.362 (2)	O12—S1	1.5511 (11)
C6—H6	0.95	O12—H12	0.84
C7—C8	1.402 (2)	O13—S1	1.4467 (12)
C7—H7	0.95	O14—S1	1.4491 (12)
C8—C9	1.372 (2)		
			
N1—C2—C3	120.19 (15)	C7—C8—H8	119.7
N1—C2—H2	119.9	O9—C9—C8	125.44 (14)
C3—C2—H2	119.9	O9—C9—C10	116.15 (13)
C4—C3—C2	119.46 (15)	C8—C9—C10	118.41 (14)
C4—C3—H3	120.3	N1—C10—C5	118.99 (13)
C2—C3—H3	120.3	N1—C10—C9	119.78 (13)
C3—C4—C5	120.84 (15)	C5—C10—C9	121.23 (14)
C3—C4—H4	119.6	C2—N1—C10	122.93 (14)
C5—C4—H4	119.6	C2—N1—H1	118.5
C6—C5—C10	118.55 (14)	C10—N1—H1	118.5
C6—C5—C4	123.86 (15)	H1W—O1W—H2W	108.3 (17)
C10—C5—C4	117.58 (14)	C9—O9—H9	109.5
C7—C6—C5	119.63 (15)	S1—O12—H12	109.5
C7—C6—H6	120.2	O11—S1—O13	112.76 (8)
C5—C6—H6	120.2	O11—S1—O14	112.44 (8)
C6—C7—C8	121.68 (14)	O13—S1—O14	112.45 (8)
C6—C7—H7	119.2	O11—S1—O12	108.35 (7)
C8—C7—H7	119.2	O13—S1—O12	106.44 (7)
C9—C8—C7	120.50 (14)	O14—S1—O12	103.72 (7)
C9—C8—H8	119.7		
			
N1—C2—C3—C4	0.0 (2)	C4—C5—C10—N1	0.9 (2)
C2—C3—C4—C5	0.5 (2)	C6—C5—C10—C9	0.1 (2)
C3—C4—C5—C6	179.92 (14)	C4—C5—C10—C9	−179.10 (13)
C3—C4—C5—C10	−0.9 (2)	O9—C9—C10—N1	−1.08 (19)
C10—C5—C6—C7	0.3 (2)	C8—C9—C10—N1	179.47 (12)
C4—C5—C6—C7	179.42 (14)	O9—C9—C10—C5	178.89 (12)
C5—C6—C7—C8	−0.2 (2)	C8—C9—C10—C5	−0.6 (2)
C6—C7—C8—C9	−0.3 (2)	C3—C2—N1—C10	0.0 (2)
C7—C8—C9—O9	−178.76 (14)	C5—C10—N1—C2	−0.4 (2)
C7—C8—C9—C10	0.6 (2)	C9—C10—N1—C2	179.54 (13)
C6—C5—C10—N1	−179.92 (13)		

### Hydrogen-bond geometry (Å, º)

**Table:**

*D*—H···*A*	*D*—H	H···*A*	*D*···*A*	*D*—H···*A*
N1—H1···O14	0.88	2.00	2.7690 (18)	145
O1*W*—H1*W*···O11^i^	0.85 (1)	1.89 (1)	2.7369 (17)	178 (1)
O1*W*—H2*W*···O14^ii^	0.85 (1)	2.03 (1)	2.8818 (19)	175 (1)
O9—H9···O13^iii^	0.84	1.81	2.6470 (16)	174
O12—H12···O1*W*	0.84	1.72	2.5529 (17)	172

Symmetry codes: (i) −*x*+1, −*y*+1, −*z*; (ii) *x*+1, *y*, *z*; (iii) −*x*+1, −*y*+1, −*z*+1.
